# The value of serum procalcitonin in the anti-infection therapy of acute stroke patients

**DOI:** 10.12669/pjms.37.4.3932

**Published:** 2021

**Authors:** Hui-ling Wang, Ying-lei Li, Xiao-fang Li, Zhi-zun Wang

**Affiliations:** 1 Hui-ling Wang, Department of Neurology, Affiliated Hospital of Hebei University, Baoding, Hebei, 071000, P. R. China; 2 Ying-lei Li, Department of Emergency, Baoding First Central Hospital, Baoding, Hebei, 071000, P. R. China; 3 Xiao-fang Li, Department of Neurology, Affiliated Hospital of Hebei University, Baoding, Hebei, 071000, P. R. China; 4 Zhi-zun Wang, Department of Cardiology, Affiliated Hospital of Hebei University, Baoding, Hebei, 071000, P. R. China

**Keywords:** Acute stroke, Procalcitonin, C-reactive protein, Post-stroke infection

## Abstract

**Objectives:**

To investigate the value of dynamic monitoring of serum procalcitonin (PCT) in anti-infective therapy of patients with acute stroke.

**Methods:**

This is a case control retrospective study of acute stroke patients conducted from July 2016 to October 2018, in the Department of Neurology, Affiliated Hospital of Hebei University, who who reached within twenty four hours. They, were selected as the study subjects who were divided into infection group and non-infection group according to the inclusion and exclusion criteria. The serum PCT and CRP levels were compared between the two groups at 24 hours, 48 hours and 72 hours. In order to judge the changes of PCT level and the infection of stroke patients, different kinds of antibiotics were used for corresponding treatment. Retrospective analysis of the cases that did not monitor PCT anti infective treatment before July 2016 were compared with the cases that monitored PCT to guide anti infective treatment after July 2016, and compared the efficacy of antibiotics.

**Results:**

The serum PCT level of patients in the infection group was significantly higher than that of patients in the noninfection group (P<0.001). For the patients whose PCT<0.5 ng/ml within 72 hour, anti-infective therapy was not administered. However, for those patients whose PCT<0.5 ng/ml and CRP rose significantly, WBC, body temperature and chest CT were closely monitored. For the patients whose PCT increased slightly (0.5 ng/ml<PCT<2.0 ng/ml), first-generation and second-generation cephalosporin or semisynthetic penicillin, such as mezlocillin, were administered. For the patients whose PCT increased moderately (5 ng/ml>PCT>2 ng/ml), mezlocillin/ sulbactam or ceftriaxone/ tazobactam was administered. For patients whose PCT increased significantly (PCT>5 ng/ml), carbapenem antibiotic or a combination of two antibiotics was administered.

**Conclusion:**

Dynamic detection of serum PCT concentration can make accurate judgment on the severity of bacterial infection in patients with acute stroke and guide the rational application of antibiotics.

## INTRODUCTION

Infection after acute stroke presents a high occurrence rate (15%-65%) clinically. Systemic immunity after cerebral ischemia is characterized by a two-phase reaction: the acute hyperinflammatory phase promotes the infiltration of brain immune cells, followed by continuous systemic immune suppression in the subacute phase, which is associated with increased mortality, poor functional prognosis, and high incidence of infection related.^1^ Thus, judging stroke, especially correctly distinguishing infection and non-infection of severe stroke patients and then adopting a series of effective treatment measures are of great clinical significance for improving the prognosis of stroke patients and enhancing the survival rate. In addition, antibiotic abuse can be avoided,^2^ and drugs can be saved. After acute stroke, patients may suffer from central fever, thus affecting clinicians’ judgment of real infection of patients. Thus, a relatively specific indicator of post-stroke infection is badly needed clinically.

Traditional infection indicators, such as hemocyte and neutrophil count, are used under restrictions due to low specificity.^3^ Some studies even indicated that by comparing the patients with infection and without infection after acute stroke and analyzing white blood cell and neutrophil count, they found that there was no obvious difference. C-reactive protein (CRP) is a product in the acute phase of inflammation, and it is often used to help diagnose bacterial infection. However, CRP increases in non-infectious inflammation, such as allergic disease, and it rises significantly in the active stage, so it lacks specificity.^4^ Serum procalcitonin (PCT), as a new indicator of systemic bacterial infection, shows high sensitivity and specificity compared with other traditional clinical inflammation indexes.^5^ This study aims to explore the clinical significance of dynamic monitoring of PCT levels on the judgment of infection and prognosis of patients with acute stroke.

## METHODS

### Ethical Approval

The study was approved by the Institutional Ethics Committee of Affiliated Hospital of Hebei University on July 1, 2018(No.HDFY-LL-2020-135), and written informed consent was obtained from all participants.

The patients with acute stroke who reached hospital within 24 hours and treated in the Department of Neurology of Affiliated Hospital of Hebei University from July 2016 to October 2018 were chosen as the study subjects. Total 832 patients met the inclusion criteria who did not require PCT for routine anti infection treatment before July 2016 were in the original diagnosis group, and 898 patients who were monitored by PCT for anti-infection treatment after July 2016 were in the existing diagnosis group. Cases with infection (including pulmonary infection, urinary tract infection, infectious diarrhea, sinusitis, etc.) within five days after the onset of the disease were included in the infection group, and the rest were included in the non infection group. Infection diagnosis conformed to the Diagnostic Criteria for Nosocomial Infection issued by the Ministry of Health of the People’s Republic of China in 2001.

### Inclusion criteria

(1) hospitalized within 24 hour of attack; (2) stroke diagnosis conformed to the standards revised in the 4^th^ National Academic Conference of Cerebrovascular Disease (including cerebral hemorrhage and cerebral infarction); (3) age ≥18 years old.

### Exclusion criteria

(1) transient ischemic attack; (2) concurrent conditions such as cardiopulmonary resuscitation, trauma, post operation, burn, shock, sunstroke, neuroendocrine neoplasm, extracorporeal circulation, liver cirrhosis, pancreatitis, mesenteric necrosis and catheter infections; applicable to any circumstance of proinflammatory factor drug release; (3) Those who arrived at the hospital were already infected (including lung infection, urinary tract infection, infectious diarrhea and nasosinusitis).

The presurvey results showed that δ=1.2, α= 0.05 (bilateral), and β = 0.2; sample size estimation formula by comparing the mean of two independent samples: n = 910. Adjust the sample size of each group according to the actual situation. A total of 898 cases in present method diagnosis group were included in this study, including 538 male cases and 360 female cases, with an average age of 62.73±12.46. APACHE II score 19.94±5.41; average duration of hospital stay was 7.86±6.42 day. The comparison of the baseline date of both groups is shown in Table-I, P>0.05. There was no significant difference between the groups.

**Table-I:** Comparison of baseline date of both groups.

Group	Infection group (N=512)	Non-infection group (N=386)	t/χ^2^	P
Age	65.1±1.92	64.83±3.16	1.584	0.114
Male (n, %)	307 (60)	231 (60)	0.001	0.972
Female (n, %)	205(40)	155(40)		
Concomitant disease (n, %)	117 (22.9)	90 (23.3)	0.027	0.870

### General information

Neurologic impairment degree was scored by the well-trained doctors in the Department of Neurology as per the National Institutes of Health Stroke Scale (NIHSS). The patients were further classified into mild group (≤6 scores), moderate group (7-14 scores) and severe group (≥15 scores) as per NIHSS. The gender, age, stroke type (cerebral hemorrhage or cerebral infarction), concomitant disease, cerebral hemorrhage and bleeding amount of all patients were recorded. Routine blood examination, routine urine examination, routine excrement examination, hepatic and renal function examination and chest radiography were conducted for the patients during hospitalization. The amount of bleeding in patients with intracerebral hemorrhage was calculated according to the hematoma seen in head CT. By measuring the length, width and height of the hematoma, and then multiplying the length by the width by the height and dividing by two, the value obtained was the total amount of blood. Infection indicator collection: The body temperatures of patients were recorded at four hour intervals. If body temperature was >37.0°C or < 35.0°C, erythrocyte sedimentation rate, chest CT and abdominal ultrasound scan were conducted. When the number of excrements increased and the excrement character changed, the excrement was cultured. When body temperature ≥38.5°C or <35.0°C, two sets of venous blood at the upper and lower limbs were collected for bacterial and fungal culture. If there was no obvious infection site, paranasal sinus CT examination was conducted.

### PCT and CRP determination

Serum PCT and CRP concentrations of patients were determined at 24 hour, 48 hour, and 72 hour, after the attack. PCT was tested by the double antibody sandwich method (Cobas e 601 Electrochemical Luminescence Automatic Immunoassay System, Roche Diagnostics Products Shanghai Limited). Serum sensitivity was ≤0.02 ng/ml. The upper limit of the normal value is 0.05 ng/ml. CRP was tested with the turbidimetry method (Cobas C 501 analysis meter, Roche Diagnostics Products Shanghai Limited). The upper limit of the normal value is 10.0 mg/L. In this study, venous blood was collected and centrifuged immediately for five minutes. Twenty microliters of serum was collected. Then, the serum was placed in a 70°C refrigerator for uniform concentration determination.

### Statistical Analysis

STATA 14.0 software was employed for data analysis. Measurement data were tested with a paired t test and expressed as ±S (mean± standard deviation). The X^2^ test or Fisher exact probability method was adopted for rate comparison. P<0.05 indicates that the difference was statistically significant.

## RESULTS

The serum PCT level of patients in the infection group was significantly higher than that of patients in the noninfection group (P<0.001), as shown in Table-II.

**Table-II:** PCT comparison of both groups.

	24h	48h	72h
PCT value ng/ml			
Infection group	3.51±2.81	3.49±2.07	3.27±2.09
Non-infection group	0.26±0.15	0.24±0.16	0.23±0.21
T value	26.696	30.773	28.465
P value	<0.001	<0.001	<0.001

The patients were grouped according to NHISS score. PCT and CRP changes within 72 hour were monitored continuously. For the patients whose PCT<0.5 ng/ml within 72 hour, anti-infective therapy was not administered. However, for those patients whose PCT<0.5 ng/ml and CRP rose significantly, WBC, body temperature and chest CT were closely monitored. For the patients whose PCT increased slightly (0.5 ng/ml<PCT<2.0 ng/ml), first-generation and second-generation cephalosporin or semisynthetic penicillin, such as mezlocillin, were administered. For the patients whose PCT increased moderately (5 ng/ml>PCT>2 ng/ml), mezlocillin/ sulbactam or ceftriaxone/ tazobactam was administered. For patients whose PCT increased significantly (PCT>5 ng/ml), carbapenem antibiotic or a combination of two antibiotics was administered.^6^

Antibiotic use comparison of the existing diagnosis group and the original diagnosis group is shown in Table-III.

**Table-III:** Antibiotic application comparison.

	Original diagnosis group n=832	Existing diagnosis group n=898	χ^2^value	P value
Application rate of first and second generation of cephalosporin (n, %)	570 (68.6)	309 (33.3)	217.573	<0.001
Application rate of third generation of cephalosporin (n, %)	201 (51.4%)	237 (25.6%)	0.447	0.504
Application rate of carbapenem (n, %)	428 (35.7%)	271 (29.2%)	90.625	<0.001
Vancomycin (n, %)	127(15.2%)	77 (8.23%)	20.780	<0.001
Application rate of antibiotics (n, %)	830(100%)	734 (79.2%)	189.301	<0.001

**Note:** P <0.05, the difference has statistical significance

## DISCUSSION

In recent years, procalcitonin (PCT) is a peptide measurable in serum which becomes elevated in response to bacterial infection. It is mostly used in lung infection, infectious shock and sepsis, etc.^7^ As a 116 amino acid residue, PCT is a protein synthesized during sepsis and inflammation. In these states, its production is stimulated by inflammatory mediators and bacterial toxins.^8^,^9^ Under bacterial infection, the patients will generate a series of reactions. PCT has been demonstrated to be ideal marker with highest accuracy for bacterial infections- allowing an early diagnosis, informing about the course and prognosis of the disease and facilitating therapeutic decisions. It can be used to distinguish between bacterial infections and non-bacterial infections, and can independently predict severe bacterial infections.^10^ According to the determination, the half-life period of PCT in serum is 20-24 hour. In normal and healthy people, serum PCT levels are very low and almost cannot be detected. For patients with serious infection, their serum PCT level will double. After general infection for four hour PCT can be detected and rises sharply after six hour. This level will be maintained within 6-24 hour .^11^,^12^ The international expert seminar pointed out that PCT-guided antibiotic management has been proven to reduce the use of antibiotics, with lower side effects and an improvement in clinical outcomes.^13^ PCT is a glycoprotein. The PCT level is low for normal people. In the case of bacterial infection, the PCT level can rise significantly, while its rise is not obvious without bacterial infection. Thus, PCT can be used to distinguish bacterial infection and nonbacterial infection.^14^ PCT will increase significantly in cases of severe infection caused by fungus, bacteria and parasite or sepsis and multiple organ failure.^15^ Usually, PCT will not rise in cases of virus infection, allergy, autoimmune disease, slight infection, partial infection and chronic infection. Bacterial endotoxin in patients can induce PCT expression. PCT can reflect the degree of patients’ inflammatory response.^16^ Furthermore, the size of the infected organ, type, bacterial species, inflammation degree and immunoreaction will influence the PCT level. Therefore, PCT is an important indicator to distinguish bacterial infection and nonbacterial infection. CRP is an acute phase reactive protein that is induced by interleukin-6 under stress states, including infection and synthesis by the liver. Determination of the serum CRP level carries certain significance for dynamic monitoring of the inflammatory response degree.^17^

The results of this study show that the serum PCT level of the infection group was significantly higher than that of the noninfection group (P < 0.05). Traditional inflammation indicators (the highest body temperature, WBC and neutrophil granulocyte classification) and CRP were not significantly different (P > 0.05). This may be related to central hyperpyrexia and irritability rise of various inflammatory factors caused by cerebral central nervous system impairment. This study shows that among patients with acute stroke, early detection of serum PCT can distinguish infection and non-infection. During dynamic monitoring of serum PCT changes, the PCT level of the infection group was higher than that of the non-infection group at all times. PCT began to rise in the early stage of infection, peaked during infection and then dropped gradually. With the infection control, PCT was close to the normal value. This does not merely show that serum PCT rise can reflect the state of continuous infection existence. In addition, dynamic changes of serum PCT can reflect infection control situations and have guiding significance for antibiotic use. According to the study of 898 patients, the PCT of patients with acute stroke rose slightly (<0.5 ng/ml). For stroke patients with PCT<0.5 ng/ml, anti-infective therapy could not be administered. For patients with a mild rise of PCT, the first and second generation of cephalosporin or semisynthetic penicillin, such as mezlocillin, could be used for anti-infective therapy. For those with moderate and severe PCT rise, active anti-infective therapy should be administered.

## CONCLUSIONS

PCT of acute stroke patients within 24 hour of attack rises mildly (0.05 ng/ml<PCT<0.25 ng/ml), slightly higher than the level of healthy persons (PCT<0.05 ng/ml). For different PCT values, different levels of antibiotic therapy should be applied. With the use of antibiotic prevalence of acute stroke patients in the ICU of the Department of Neurology declined to 79.2% from almost 100%. The antibiotic use of carbapenem declined to 29.2% from 35.7%. The infection rate of multiple resistant bacteria declined to 8.23% from 15.2%. For the acute stroke patients in the general wards, the antibiotic use decreased to 12.19% from 20.12%. It can be seen from the above data that if this program is further promoted, it will be of great importance for rational use of antibiotics and reduction in drug-resistant bacteria.^18^

### Innovations and limitations of the study

Serum PCT is a specific indicator to differentiate whether acute stroke patients are infected in the early stage. It is obviously superior to traditional inflammation indicators (such as body temperature and WBC) and CRP.^19^ However, it is worth noting that the specificity and sensitivity of serum PCT in predicting the death of patients with infection are not 100%, so traditional WBC and CRP should be combined in clinical application to improve the accuracy. Serum PCT provides beneficial evidence for clinicians to diagnose bacterial infection early so that clinicians can guide patients to take drugs, which avoids abuse of antibiotics and blind change more potent and expensive antibiotics. In addition, dynamic monitoring of serum PCT concentration changes can help clinicians make accurate judgments of bacterial infection severity, better judge prognosis and observe curative effects. The generalization of this program will greatly help improve the healthcare level of our province.^20^ With rational use of antibiotics drug-resistant strains can be reduced. Moreover, patients’ economic burden can be relieved, and medical resources can be rationally optimized.

### Authors’ Contributions:

**HLW &** **XFL:** Designed this study and prepared this manuscript, and are responsible and accountable for the accuracy or integrity of the work.

**YLL:** Collected and analyzed clinical data.

**ZZW:** Significantly revised this manuscript.
